# Clausenlanins A and B, Two Leucine-Rich Cyclic Nonapeptides from *Clausena lansium*

**DOI:** 10.1007/s13659-017-0133-y

**Published:** 2017-06-13

**Authors:** Shai-Ping Hu, Wei-Wu Song, Si-Meng Zhao, Ning-Hua Tan

**Affiliations:** 1 School of Traditional Chinese Pharmacy and State Key Laboratory of Natural Medicines, China Pharmaceutical University, Nanjing, 211198 Jiangsu People’s Republic of China; 2 State Key Laboratory of Phytochemistry and Plant Resources in West China, Kunming Institute of Botany, Chinese Academy of Sciences, Kunming, 650201 Yunnan People’s Republic of China; 3 School of Chemistry & Chemical Engineeing, Zhoukou Normal University, Zhoukou, 466001 Henan People’s Republic of China

**Keywords:** *Clausena lansium*, Cyclopeptides, Clausenlanin A, Clausenlanin B

## Abstract

**Abstract:**

Two new cyclic nonapeptides, named clausenlanins A (**1**) and B (**2**), were isolated from the roots and rhizomes of *Clausena lansium.* Their structures were elucidated as cyclo-(Gly^1^-l-Leu^2^-l-Ile^3^-l-Leu^4^-l-Leu^5^-l-Leu^6^-l-Leu^7^-l-Leu^8^-l-Leu^9^) (**1**) and cyclo-(Gly^1^-l-Leu^2^-l-Val^3^-l-Leu^4^-l-Leu^5^-l-Leu^6^-l-Leu^7^-l-Leu^8^-l-Leu^9^) (**2**) respectively on the basis of extensive spectroscopic analysis, particularly 2D NMR spectra taken at the temperature of 338 or 303 K and MS.

**Graphical Abstract:**

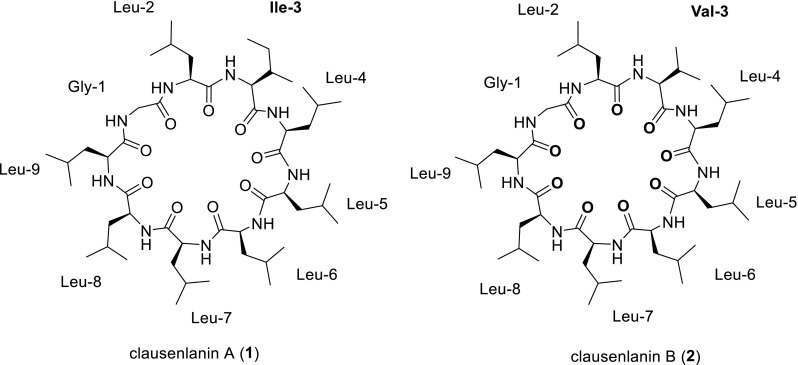

**Electronic supplementary material:**

The online version of this article (doi:10.1007/s13659-017-0133-y) contains supplementary material, which is available to authorized users.

## Introduction

**Fig. 1:**
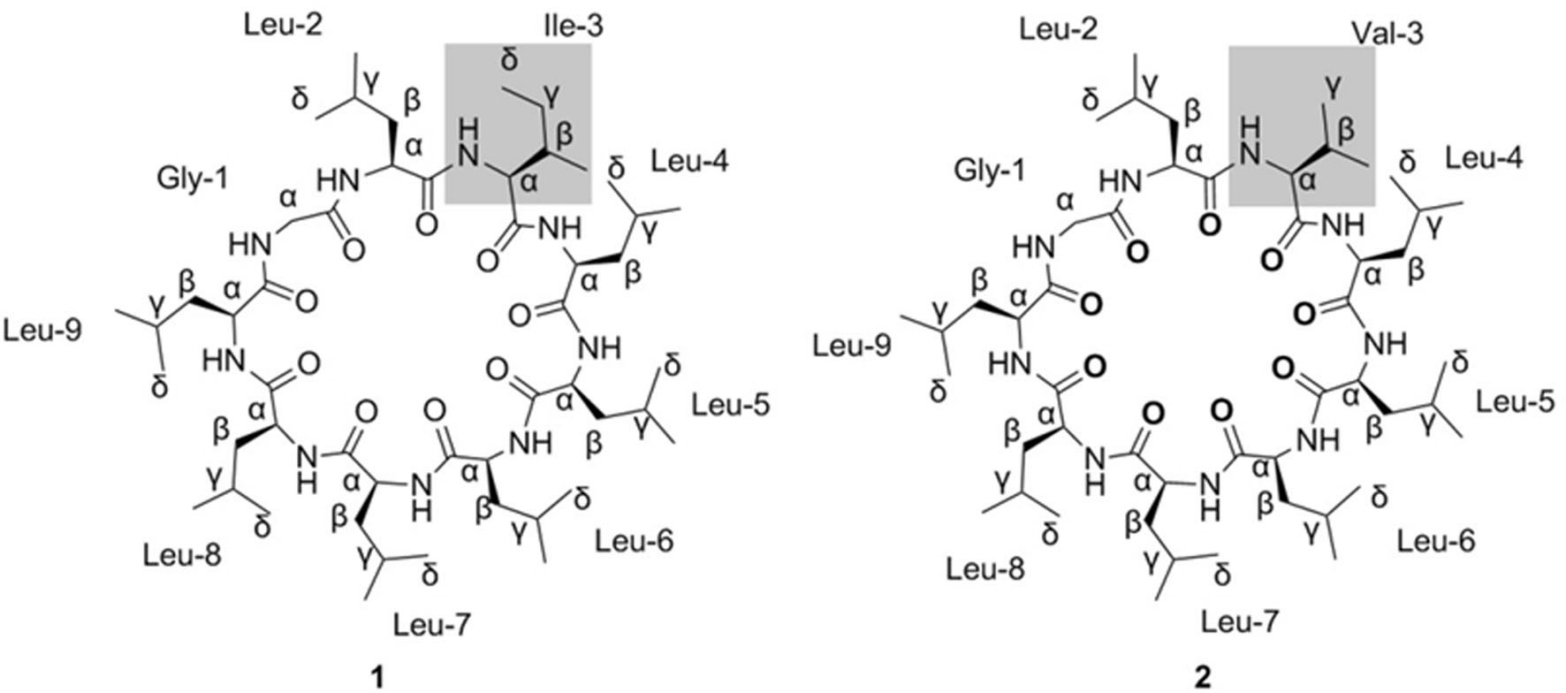
Structures of compounds **1** and **2** from *C. lansium*

About 30 species of *Clausena* (Rutaceae) are widely distributed in the world, and 10 of them exist in China. *Clausena lansium* (Lour.) Skeels is a fruit tree and distributes widely in south of China [1]. Its leaves and roots have been used as a folk herb for the treatment of cough, asthma, dermatological disease, viral hepatitis, and gastro-intestinal disease; and its seeds for treating acute and chronic gastrointestinal inflammation, and ulcer [2]. Caryophyllaceae-type cyclopeptides (CPs), carbazole alkaloids, coumarins, amides, and terpenoids have been isolated from *C. lansium* [3–8]. Among them, CPs are formed with the peptide bonds of protein or non-proten α-amino acid residues, which are homomonocyclopeptides with mainly five to twelve α-amino acid residues [9]. During this work, two new cyclic nonapeptides, named clausenlanins A (**1**) and B (**2**) (Fig. 1), were isolated from the roots and rhizomes of *C. lansium*. Because the ^1^H NMR signals are weak and severely overlapped taken at room temperature, variable temperature NMR experiments were performed [10]. In this paper, their separation and structure elucidation are described.

## Results and Discussion

Clausenlanin A (**1**) was obtained as an amorphous solid. Its molecular formula was shown as C_50_H_91_N_9_O_9_ by its negative HRESIMS ([M−H]^−^, 960.6876, calcd 960.6867), indicating the 10° of unsaturation. The IR spectrum exhibited the absorption bands at 3429 and 1661 cm^–1^ ascribable to NH and CO groups. The ^1^H and ^13^C NMR spectra of **1** in C_5_D_5_N (Table 1) displayed the characteristic signals of typical CPs.

**Table 1:** NMR data of compounds **1** and **2**

1*				**2** ^#^			
Residue	Position	*δ*_H_	*δ*_C_	Residue	Position	*δ*_H_	*δ*_C_
Gly-1	α	4.51 (d, 5.1)	44.8 (t)	Gly-1	α	4.57 (overlap)	44.6 (t)
		3.85 (dd, 5.1, 16.3)				3.91 (dd, 5.2, 16.4)	
	NH	8.75 (overlap)			NH	9.03 (br.s)	
	C=O		171.1 (s)		C=O		171.2 (s)
Leu-2	α	4.77 (d, 7.0)	53.7 (d)	Leu-2	α	4.87 (br.s)	53.3 (d)
	β	1.93–2.21 (overlap)	40.6 (t)		β	1.93 (overlap)	40.4 (t)^a^
						2.07 (overlap)	
	γ	1.87–1.99 (overlap)	25.8 (d)^a^		γ	1.89–2.00 (overlap)	25.8 (d)^b^
	δ	0.93–1.06 (overlap)	23.7 (q)^b^		δ	0.86–1.03 (overlap)	23.4 (q)^c^
		0.91–1.02 (overlap)	22.3 (q)^c^			0.86–1.03 (overlap)	22.0 (q)^d^
	NH	8.23 (d, 7.0)			NH	8.42 (br.s)	
	C=O		175.6 (s)		C=O		174.7 (s)^e^
Ile-3	α	4.46 (overlap)	61.2 (d)	Val-3	α	4.42 (br.s)	62.6 (d)
	β	2.30 (overlap)	36.7 (d)		β	2.57 (m)	30.6 (d)
	γ	1.39 (m)	26.7 (t)		γ	1.18 (d, 4.4)	20.3 (q)
		1.87 (overlap)				1.19 (d, 4.4)	20.2 (q)
	Me γ	1.18 (d, 6.7)	16.6 (q)				
	Me δ	0.92 (overlap)	11.5 (q)				
	NH	8.72 (overlap)			NH	9.22 (overlap)	
	C=O		173.9 (s)		C=O		173.9 (s)
Leu-4	α	4.58 (overlap)	54.5 (d)	Leu-4	α	4.65 (overlap)	54.6 (d)
	β	1.93–2.21 (overlap)	40.0 (t)^d^		β	2.07 (overlap)	39.9 (t)^a^
	γ	1.87–1.99 (overlap)	25.9 (d)^a^		γ	1.89–2.00 (overlap)	25.7 (d)^b^
	δ	0.93–1.06 (overlap)	23.6 (q)^b^		δ	0.86–1.03 (overlap)	23.6 (q)^c^
		0.91–1.02 (overlap)	22.3 (q)^c^			0.86–1.03 (overlap)	22.0 (q)^d^
	NH	8.73 (overlap)			NH	9.23 (overlap)	
	C=O		173.5 (s)		C=O		173.8 (s)^e^
Leu-5	α	4.81 (d, 7.2)	54.0 (d)	Leu-5	α	4.93 (br.s)	53.7 (d)
	β	1.93–2.21 (overlap)	40.6 (t)		β	2.17 (overlap)	40.4 (t)^a^
						2.31 (overlap)	
	γ	1.87–1.99 (overlap)	25.5 (d)^a^		γ	1.89–2.00 (overlap)	25.6 (d)^b^
	δ	0.93–1.06 (overlap)	23.8 (q)^b^		δ	0.86–1.03 (overlap)	23.8 (q)^c^
		0.91–1.02 (overlap)	22.6 (q)^c^			0.86–1.03 (overlap)	22.4 (q)^d^
	NH	8.10 (d, 7.2)			NH	8.35 (br.s)	
	C=O		174.4 (s)		C=O		174.7 (s)^e^
Leu-6	α	4.59 (overlap)	54.8 (d)	Leu-6	α	4.66 (overlap)	54.5 (d)
	β	1.93–2.21 (overlap)	40.3 (t)^d^		β	2.07 (overlap)	39.8 (t)^a^
						2.28 (overlap)	
	γ	1.87–1.99 (overlap)	25.8 (d)^a^		γ	1.89–2.00 (overlap)	25.2 (d)^b^
	δ	0.93–1.06 (overlap)	23.7 (q)^b^		δ	0.86–1.03 (overlap)	23.4 (q)^c^
		0.91–1.02 (overlap)	22.3 (q)^c^			0.86–1.03 (overlap)	22.0 (q)^d^
	NH	8.52 (d, 6.3)			NH	8.85 (br.s)	
	C=O		174.1 (s)		C=O		174.1 (s)^e^
Leu-7	α	4.46 (overlap)	54.9 (d)	Leu-7	α	4.50 (br.s)	54.7 (d)
	β	1.93–2.21 (overlap)	40.1 (t)		β	2.28 (overlap)	39.7 (t)^a^
						2.36 (br.s)	
	γ	1.87–1.99 (overlap)	26.0 (d)^a^		γ	1.89–2.00 (overlap)	25.7 (d)^b^
	δ	0.93–1.06 (overlap)	23.5 (q)^b^		δ	0.86–1.03 (overlap)	23.7 (q)^c^
		0.91–1.02 (overlap)	22.1 (q)^c^			0.86–1.03 (overlap)	21.8 (q)^d^
	NH	8.46 (d, 6.2)			NH	8.76 (br.s)	
	C = O		174.7 (s)		C = O		175.7 (s)^e^
Leu-8	α	4.68 (dd, 6.3, 7.9)	54.7 (d)	Leu-8	α	4.79 (br.s)	54.1 (d)
	β	1.93–2.21 (overlap)	40.3 (t)		β	2.07 (overlap)	40.0 (t)^a^
						2.19 (overlap)	
	γ	1.87–1.99 (overlap)	25.8 (d)^a^		γ	1.89–2.00 (overlap)	25.6 (d)^b^
	δ	0.93–1.06 (overlap)	23.7 (q)^b^		δ	0.86–1.03 (overlap)	23.7 (q)^c^
		0.91–1.02 (overlap)	22.3 (q)^c^			0.86–1.03 (overlap)	22.0 (q)^d^
	NH	8.36 (d, 6.3)			NH	8.61 (br.s)	
	C=O		174.5 (s)		C=O		174.6 (s)^e^
Leu-9	α	4.57 (overlap)	53.6 (d)	Leu-9	α	4.56 (overlap)	53.6 (d)
	β	1.93–2.21 (overlap)	40.1 (t)^d^		β	2.17 (overlap)	40.4 (t)^a^
						2.28 (overlap)	
	γ	1.87–1.99 (overlap)	25.9 (d)^a^		γ	1.89–2.00 (overlap)	25.7 (d)^b^
	δ	0.93–1.06 (overlap)	23.5 (q)^b^		δ	0.86–1.03 (overlap)	23.8 (q)^c^
		0.91–1.02 (overlap)	22.2 (q)^c^			0.86–1.03 (overlap)	22.0 (q)^d^
	NH	8.91 (br.s)			NH	9.38 (overlap)	
	C=O		173.9 (s)		C=O		173.5 (s)^e^

J values given in Hz in parentheses

^a, b, c, d, e^ Chemical shifts can be exchanged with each other in the column

* In C_5_D_5_N, 338 K, 400 MHz for ^1^H and 100 MHz for ^13^C

^#^In C_5_D_5_N, 303 K, 800 MHz for ^1^H and 200 MHz for ^13^C

**Fig. 2:**
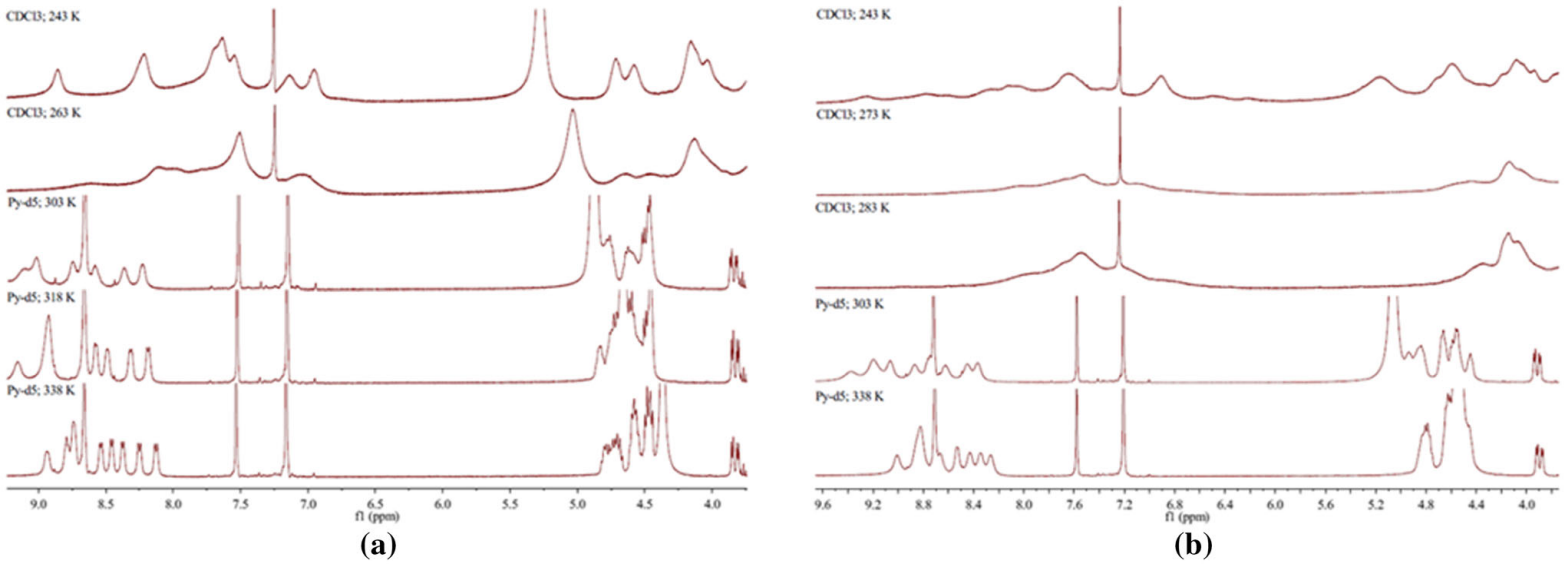
^1^H NMR of compounds **1** (**a**) and **2** (**b**) at different temperatures

**Fig. 3:**
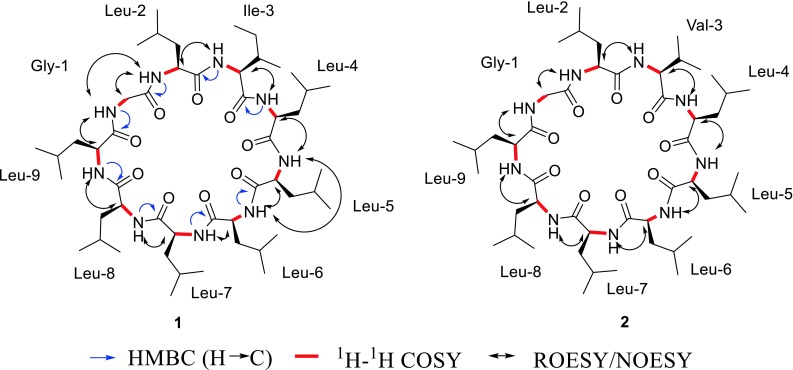
Key HMBC, ^1^H-^1^H COSY, and ROESY/NOESY correlations of **1** and **2**

The ^1^H NMR signals of the amino acid residues of **1**, especially the signals of NH and α-H, were severely overlapped taken at room temperature. The significant improvement of the ^1^H NMR signals was observed by increasing the temperatures from 243 to 338 K. Finally a well-resolved ^1^H NMR spectrum with sharp proton signals (Fig. 2a) was obtained at 338 K in pyridine-d_5_. Then the assignment of the ^1^H NMR signals of the amino acid residues was obtained by analyzing the ^1^H-^1^H COSY spectrum, particularly amide proton NH and α-H signals. The corresponding ^13^C NMR assignments were determined on the basis of the HSQC and HMBC experiments, particularly α-C signals (Table 1). The ^1^H-NMR spectrum of **1** showed the presence of nine NH (*δ*_H_ 8.91, 8.75, 8.73, 8.72, 8.52, 8.46, 8.36, 8.23, 8.10) and ten α-H (*δ*_H_ 4.81, 4.77, 4.68, 4.59, 4.58, 4.57, 4.51, 4.46, 4.46, 3.85), respectively. The ^13^C-NMR spectrum of **1** displayed nine carbonyl CO signals at *δ*_C_ 175.6, 174.7, 174.5, 174.4, 174.1, 173.9, 173.9, 173.5, 171.1, eight α-CH signals at *δ*_C_ 61.2, 54.9, 54.8, 54.7, 54.5, 54.0, 53.7, 53.6, one α-CH_2_ signal at *δ*_C_ 44.8. These data indicated that **1** might be a cyclic nonapeptide. Analysis of the HSQC, HMBC and COSY spectra revealed that **1** consisted of one glycine (*δ*_H_ 4.51 and 3.85 (α-H_2_), 8.75 (NH); *δ*_C_ 171.1 (CO), 44.8 (α-CH_2_)), and one isoleucine (*δ*_H_ 4.46 (α-CH), 2.30 (β-CH), 1.39 and 1.87 (γ-CH_2_), 1.18 (γ-CH_3_), 0.92 (δ-CH_3_), 8.72 (NH); *δ*_C_ 173.9 (CO), 61.2 (α-CH), 36.7 (β-CH), 26.7 (γ-CH_2_), 16.6 (γ-CH_3_), 11.5 (δ-CH_3_)). The remaining signals mentioned-above of seven NH and seven α-H signals, seven CO and seven α-C signals, and other signals including seven methylenes at *δ*_C_ 40.0–40.6, seven methines at *δ*_C_ 25.5–26.0, two kinds of fourteen methyls at *δ*_C_ 23.5–23.8 and at *δ*_C_ 22.1–22.6, indicated that **1** contained other seven leucines. Therefore **1** consisted of seven leucines, one glycine, and one isoleucine (Table 1; Fig. 3).

The sequence of the nine amino acid residues in **1** was determined by analyzing the ROESY correlations between the α-H of one amino acid residue and the amide proton NH of the next amino acid residue (Fig. 3). The ROESY correlations of Gly^1^-αH/Leu^2^-NH, Leu^2^-αH/Ile^3^-NH, Ile^3^-αH/Leu^4^-NH, Leu^4^-αH/Leu^5^-NH, Leu^5^-αH/Leu^6^-NH, Leu^6^-αH/Leu^7^-NH, Leu^7^-αH/Lue^8^-NH, Leu^8^-αH/Leu^9^-NH, Leu^9^-αH/Gly^1^-NH indicated that the structure of **1** is cyclo-(Gly^1^-Leu^2^-Ile^3^-Leu^4^-Leu^5^-Leu^6^-Leu^7^-Leu^8^-Leu^9^). This sequence of **1** was confirmed by the fragment ion peaks at 962.96 [M+H]^+^, 849.83 [M+H−113]^+^, 736.72 [M+H−2*113]^+^, 623.64 [M+H−3*113]^+^, 510.52 [M+H− 4*113]^+^, 453.49 [M+H−4*113−57]^+^, 340.43 [M+H−4*113–57−113]^+^, 227.29 [M+H−4*113–57−2*113]^+^ in the positive ESIMSMS.

The absolute configuration of the amino acids of **1** was determined using the advanced Marfey’s method and LC–MS analysis [11, 12]. The results indicated that the absolute configurations of the amino acid residues (Leu and Ile) in **1** were the l-configuration (Table S1; Fig. 3). Therefore the structure of **1** is determined as cyclo-(Gly^1^-l-Leu^2^-l-Ile^3^-l-Leu^4^-l-Leu^5^-l-Leu^6^-l-Leu^7^-l-Leu^8^-l-Leu^9^).

Clausenlanin B (**2**) was obtained as an amorphous solid. Its molecular formula was shown as C_49_H_89_N_9_O_9_ by its negative HRESIMS ([M−H]^−^, 946.6723, calcd 946.6710), indicating the 10° of unsaturation. The IR spectrum exhibited the absorption bands at 3430 and 1661 cm^–1^ ascribable to NH and CO groups. The ^1^H and ^13^C NMR spectra of **2** in C_5_D_5_N (Table 1) displayed the characteristic signals of typical CPs.

The ^1^H NMR signals of the amino acid residues of **2**, especially the signals of NH and α-H, were severely overlapped taken at room temperature. The significant improvement of the ^1^H NMR signals was observed by increasing the temperatures from 243 to 338 K. Finally a well-resolved ^1^H NMR spectrum with sharp proton signals (Fig. 2b) was obtained at 303 K in pyridine-d_5_. After compared all data of **2** with those of **1**, the results indicated that **2** and **1** are very similar, and **2** might also be a cyclic nonapeptide too. The only difference is to be replaced the isoleucine residue in **1** by valine residue in **2**. The assignment of the ^1^H and ^13^C NMR signals of the valine residue was obtained by analyzing the HSQC, HMBC and COSY spectra, i.e. *δ*_H_ 4.42 (α-CH), 2.57 (β-CH), 1.18 and 1.19 (2*γ-CH_3_), 9.22 (NH); *δ*_C_ 173.9 (CO), 62.6 (α-CH), 30.6 (β-CH), 20.3 and 20.2 (2*γ-CH_3_). Therefore **2** consisted of seven leucines, one glycine, and one valine (Table 1; Fig. 3).

The sequence of the nine amino acid residues in **2** was determined by analyzing NOESY correlations between the α-H of one amino acid residue and the amide proton NH of the next amino acid residue (Fig. 3). The NOESY correlations of Gly^1^-αH/Leu^2^-NH, Leu^2^-αH/Val^3^-NH, Val^3^-αH/Leu^4^-NH, Leu^4^-αH/Leu^5^-NH, Leu^5^-αH/Leu^6^-NH, Leu^6^-αH/Leu^7^-NH, Leu^7^-αH/Lue^8^-NH, Leu^8^-αH/Leu^9^-NH, Leu^9^-αH/Gly^1^-NH indicated that the structure of **2** is cyclo-(Gly^1^-Leu^2^-Val^3^-Leu^4^-Leu^5^-Leu^6^-Leu^7^-Leu^8^-Leu^9^). This sequence of **2** was confirmed by the fragment ion peaks at 948.86 [M+H]^+^, 835.70 [M+H−113]^+^, 722.56 [M+H−2*113]^+^, 609.54 [M+H−3*113]^+^, 496.39 [M+H−4*113]^+^, 383.41 [M+H−5*113]^+^, 270.24 [M+H−6*113]^+^ in the positive ESIMSMS.

The absolute configuration of **2** was determined using the advanced Marfey’s method and LC–MS analysis too [11, 12]. The results indicated that the absolute configurations of the amino acid residues (Leu and Val) in **2** were the l-configuration (Table S1; Fig. 3). Therefore the structure of **2** is determined as cyclo-(Gly^1^-l-Leu^2^-l-Val^3^-l-Leu^4^-l-Leu^5^-l-Leu^6^-l-Leu^7^-l-Leu^8^-l-Leu^9^).

## Experimental

### General Experimental Procedures

Optical rotations were obtained on a Jasco P-1020 polarimeter. IR spectra were measured on a Tensor 27 spectrometer with KBr pellets. UV spectra were obtained using a Shimadzu UV-2401PC spectrophotometer. 1D and 2D NMR spectra were performed on a Bruker AM-400 (^1^H: 400 MHz, ^13^C: 100 MHz) or Bruker AVANCE III-800 (^1^H: 800 MHz, ^13^C: 200 MHz). Chemical shifts were expressed in ppm with reference to the solvent signals. Mass spectra were measured on a Waters XEVO-TQD spectrometer or an Agilent 1290 UPLC/6540 Q-TOF spectrometer. Analytical or semi-preparative HPLC was performed on Agilent 1100 apparatus equipped with a UV detector and a SunFire OBD (Waters, 1.9 × 25 cm, 5 μm). Column chromatography was performed with silica gel (100–200 mesh and 200–300 mesh, Qingdao Yu-Min-Yuan Chemical Co. Ltd., Qingdao, P.R. China), MCI gel (CHP-20P, 70–150 μm, Mitsubishi Chemical Co., Japan) or Lichroprep RP-18 gel (40–63 mm, Merck, Darmstadt, Germany). Fractions were monitored by TLC (GF254, Qingdao Yu-Min-Yuan Chemical Co. Ltd., Qingdao, P.R. China), and the orange spots were visualized on the plate by spraying with 2% ninhydrin reagent, after hydrolyzed in an incubator (110 °C) for 30 min by concentrated HCl [13].

### Plant Material

The roots and rhizomes of *C. lansium* were collected in Hekou, Yunnan Province, P. R. China, in September 2010, and identified by Prof. Yu-Min Shui, Kunming Institute of Botany, Chinese Academy of Sciences. A voucher specimen (No. 0599043) was deposited at the Herbarium of Kunming Institute of Botany, Chinese Academy of Sciences.

### Extraction and Isolation

Air dried, powdered roots and rhizomes of *C. lansium* (27 kg) were extracted and refluxed with MeOH for three times each for 4 h (MeOH, 3*50 L). The extract was evaporated under reduced pressure to yield a dark brown residue (0.9 kg). The residue was suspended in MeOH/H_2_O (7:3, 3 L) and then partitioned with EtOAc (3*2 L). After removing solvent, the EtOAc-soluble part (406 g) was fractionated by silica gel (200–300 mesh) column chromatograph (CC) and eluted with CHCl_3_/MeOH (30:1–4:1) to afford six fractions (Fr.1–Fr.6), on the basis of TLC detection.

Fr.6 (77 g) was subjected to silica gel CC (CHCl_3_/acetone 15:1–7:3) to afford Fr.6.1–Fr.6.4. Fr.6.2 (14.3 g) was subjected to silica gel CC (PE/acetone 5:1), MPLC with MCI (MeOH/H_2_O 10:90–60:40), and MPLC with RP-18 (MeOH/H_2_O 5:95–70:30). Then, the fractions was further purified by silica gel CC (CHCl_3_/MeOH 50:1), subsequently to afford **1** (308 mg) and **2** (47 mg).

### Clausenlanin A (**1**)

Amorphous powder; [*α*]_D_^20.7^ –154.6 (*c* 0.10, MeOH); UV (MeOH) λ_max_ (log *ε*) 203.0 (4.61) nm; CD (MeOH) 203 (Δ*ε*–30.8); IR (KBr) ν_max_ 3429, 2960, 1661, 1528, 584 cm^–1^; ^1^H (400 MHz) and ^13^C (100 MHz) NMR data, see Table 1; positive ESIMSMS *m/z* 962.96 [M+H]^+^, 849.83 [M+H−113]^+^, 736.72 [M+H−2*113]^+^, 623.64 [M+H−3*113]^+^, 510.52 [M+H−4*113]^+^, 453.49 [M+H−4*113−57]^+^, 340.43 [M+H−4*113−57−113]^+^, 227.29 [M+H−4*113−57−2*113]^+^; negative HRESIMS *m/z* 960.6876 [M−H]^−^, calcd for C_50_H_91_N_9_O_9_, 960.6867.

### Clausenlanin B (**2**)

Amorphous powder; [*α*]_D_^20.6^ –73.69 (*c* 0.10, MeOH); UV (MeOH) λ_max_ (log *ε*) 202.8 (4.40) nm; CD (MeOH) 203 (Δ*ε*–26.1); IR (KBr) ν_max_ 3430, 2960, 1661, 1527, 584 cm^–1^; ^1^H (800 MHz) and ^13^C (200 MHz) NMR data, see Table 1; positive ESIMSMS *m/z* 948.86 [M+H]^+^, 835.70 [M+H−113]^+^, 722.56 [M+H−2*113]^+^, 609.54 [M+H−3*113]^+^, 496.39 [M+H−4*113]^+^, 383.41 [M+H−5*113]^+^, 270.24 [M+H−6*113]^+^; negative HRESIMS *m/z* 946.6723 [M−H]^−^, calcd for C_49_H_89_N_9_O_9_, 946.6710.

### Advanced Marfey’s Method [11, 12]

The cyclic peptide (about 1.0 mg each) was dissolved in 6 N HCl (1 mL) and heated at 110 °C for 24 h. The hydrolyzate was evaporated to dryness, and the residue was re-dissolved in 100 μL of acetone. To each a half portion (50 μL) were added 20 μL of NaHCO_3_ (1 M) and 100 μL of N^α^-(5-Fluoro-2,4-dinitrophenyl)-l-leucinamide (l-FDLA, 1% in acetone) or 50 μL of N^α^-(5-Fluoro-2,4-dinitrophenyl)-l-leucinamide and 50 μL of N^α^-(5-Fluoro-2,4-dinitrophenyl)-d-leucinamide (mixture of l-FDLA and d-FDLA, 1% in acetone), and the mixture was heated at 45 °C for 1.5 h. Reaction was cooled to room temperature, and then acidified with 2 N HCl (10 μL), dried and dissolved in 50% aqueous MeCN. 5 μL of each solution of FDLA derivatives were analyzed by LC/MS.

The analysis of the l- and d, l-FDLA (mixture of d- and l-FDLA) derivatives was performed using an Waters Sunfire C_18_ column (4.6*150 mm, 5 μm) maintained at 30 °C. Acetonitrile—0.1% HCOOH/H_2_O was used as the mobile phase under a linear gradient elution mode (acetonitrile, 28–60%, 50 min (compound **1**); acetonitrile, 35–60%, 50 min (compound **2**)) at a flow rate of 1 mL/min. A Waters Xevo-TQD mass spectrometer was used for detection in ESI^−^ mode. The capillary voltage was kept at 2.5 kV, and the ion source at 450 °C. Nitrogen gas was used as a sheath gas at 650 L/h. A mass range of m/z 100–2000 was scanned in 0.2 s.

## Electronic supplementary material

Below is the link to the electronic supplementary material.

Supplementary material 1 (DOC 4397 kb)
