# Supplementary material for: Clausenlanins A and B, Two Leucine-Rich Cyclic Nonapeptides from Clausena lansium
Source: Nat Prod Bioprospect. 2017 Jun 13;7(4):307–13. doi: 10.1007/s13659-017-0133-y (PMC5507809; doi:10.1007/s13659-017-0133-y)
Supplement: Supplementary file 1 — Supplementary material 1 (DOC 4397 kb) [file 13659_2017_133_MOESM1_ESM.doc]

**Supplementary Information**

**Clausenlanins A and B, Two Leucine-rich Cyclic Nonapeptides from *Clausena lansium***

Shai-Ping Hu^a,b,†^, Wei-Wu Song^b,c,†^, Si-Meng Zhao^b^, Ning-Hua Tan^a,b,*^

^a^ *School of Traditional Chinese Pharmacy and State Key Laboratory of Natural Medicines, China Pharmaceutical University, Nanjing 211198, Jiangsu, People’s Republic of China*

^b^ *State Key Laboratory of Phytochemistry and Plant Resources in West China, Kunming Institute of Botany, Chinese Academy of Sciences, Kunming 650201, Yunnan, People’s Republic of China*

*^c^* *School of Chemistry & Chemical Engineeing, Zhoukou Normal University, Zhoukou 466001, Henan, People’s Republic of China*

† These authors contributed equally to this work.

*Correspondence: nhtan@cpu.edu.cn (N.-H.Tan)

Contents

| Figure S1. ^1^H NMR spectrum of clausenlanin A (**1**) | 3 |
| --- | --- |
| Figure S2. ^13^C NMR spectrum of clausenlanin A (**1**) | 4 |
| Figure S3. HSQC spectrum of clausenlanin A (**1**) | 5 |
| Figure S4. ^1^H-^1^H COSY spectrum of clausenlanin A (**1**) | 6 |
| Figure S5. HMBC spectrum of clausenlanin A (**1**) | 7 |
| Figure S6. ROESY spectrum of clausenlanin A (**1**) | 8 |
| Figure S7. ESIMSMS spectrum of clausenlanin A (**1**) | 9 |
| Figure S8. HRESIMS spectrum of clausenlanin A (**1**) | 10 |
| Figure S9. IR spectrum of clausenlanin A (**1**) | 11 |
| Figure S10. UV spectrum of clausenlanin A (**1**) | 12 |
| Figure S11. CD spectrum of clausenlanin A (**1**) | 13 |
| Figure S12. [α]^D^ spectrum of clausenlanin A (**1**) | 14 |
| Figure S13. ^1^H NMR spectrum of clausenlanin B (**2**) | 15 |
| Figure S14. ^13^C NMR spectrum of clausenlanin B (**2**) | 16 |
| Figure S15. HSQC spectrum of clausenlanin B (**2**) | 17 |
| Figure S16. ^1^H-^1^H COSY spectrum of clausenlanin B (**2**) at 65 °C | 18 |
| Figure S17. ^1^H-^1^H COSY spectrum of clausenlanin B (**2**) at 30 °C | 19 |
| Figure S18. HMBC spectrum of clausenlanin B (**2**) | 20 |
| Figure S19. NOESY spectrum of clausenlanin B (**2**) | 21 |
| Figure S20. ESIMSMS spectrum of clausenlanin B (**2**) | 22 |
| Figure S21. HREIMS spectrum of clausenlanin B (**2**) | 23 |
| Figure S22. IR spectrum of clausenlanin B (**2**) | 24 |
| Figure S23. UV spectrum of clausenlanin B (**2**) | 25 |
| Figure S24. CD spectrum of clausenlanin B (**2**) | 26 |
| Figure S25. [α]^D^ spectrum of clausenlanin B (**2**) | 27 |
| Table S1. Determination of the absolute configurations of amino acid residues in **1** and **2** by the advanced Marfey’s method | 28 |

**Figure S1**. ^1^HNMR spectrum of clausenlanin A (**1**)


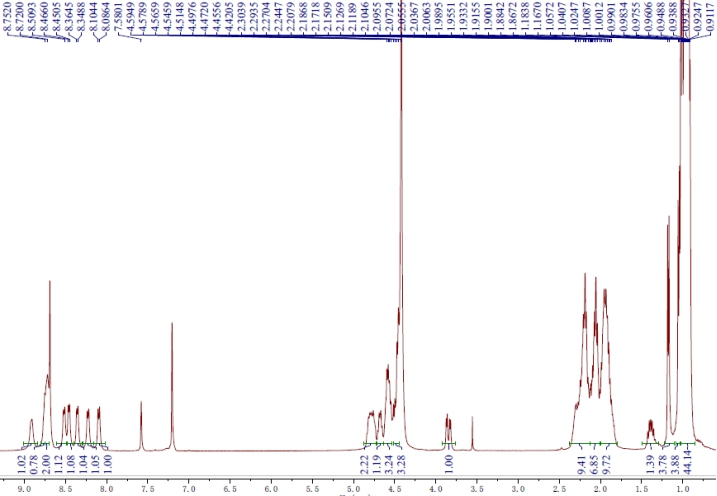


**Figure S2**. ^13^C NMR spectrum of clausenlanin A (**1**)


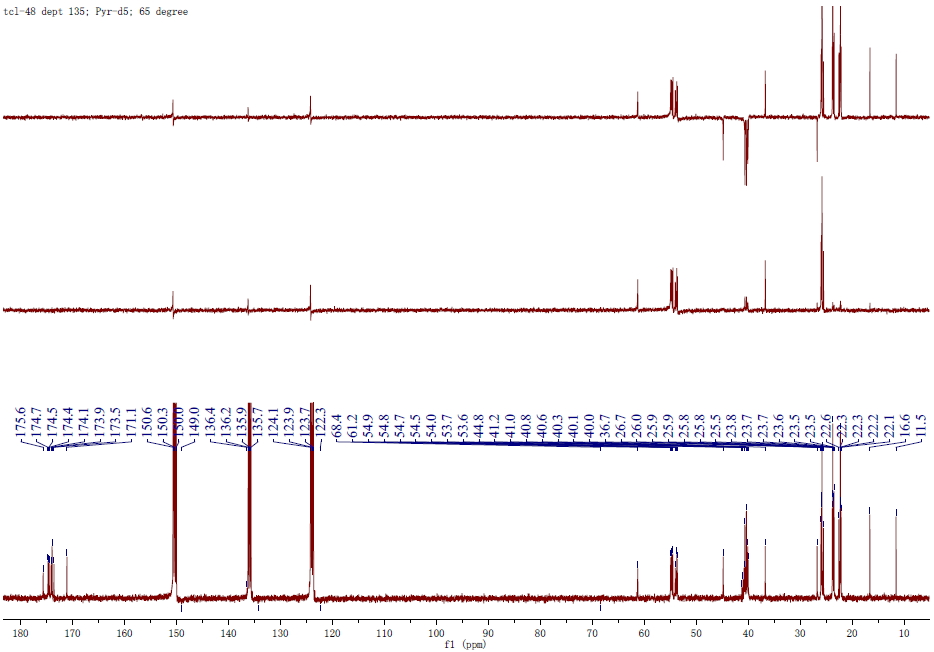


**Figure S3**. HSQC spectrum of clausenlanin A (**1**)


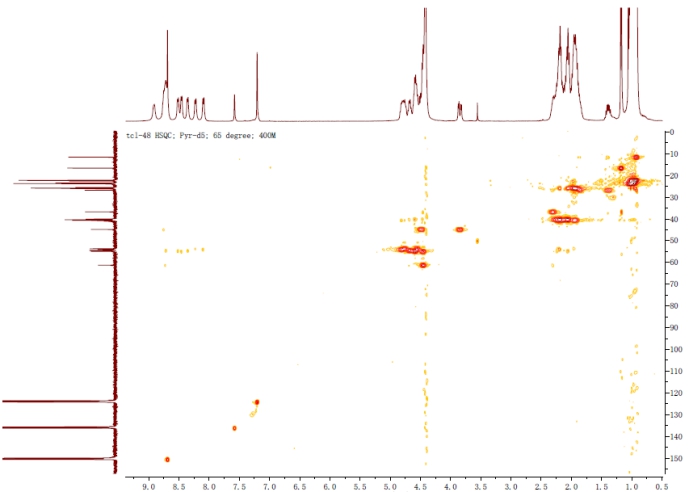


**Figure S4**. ^1^H-^1^H COSY spectrum of clausenlanin A (**1**)


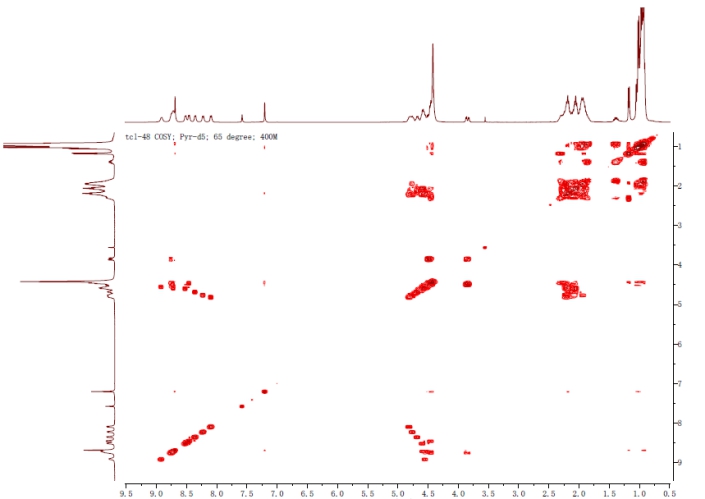


**Figure S5**. HMBC spectrum of clausenlanin A (**1**)


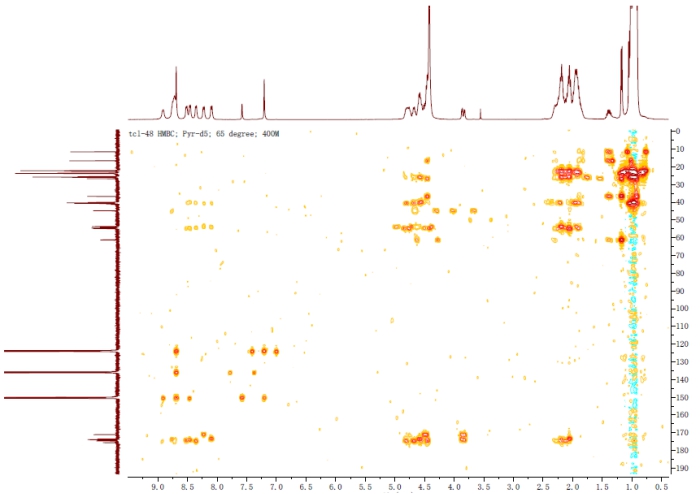


**Figure S6**. ROESY spectrum of clausenlanin A (**1**)


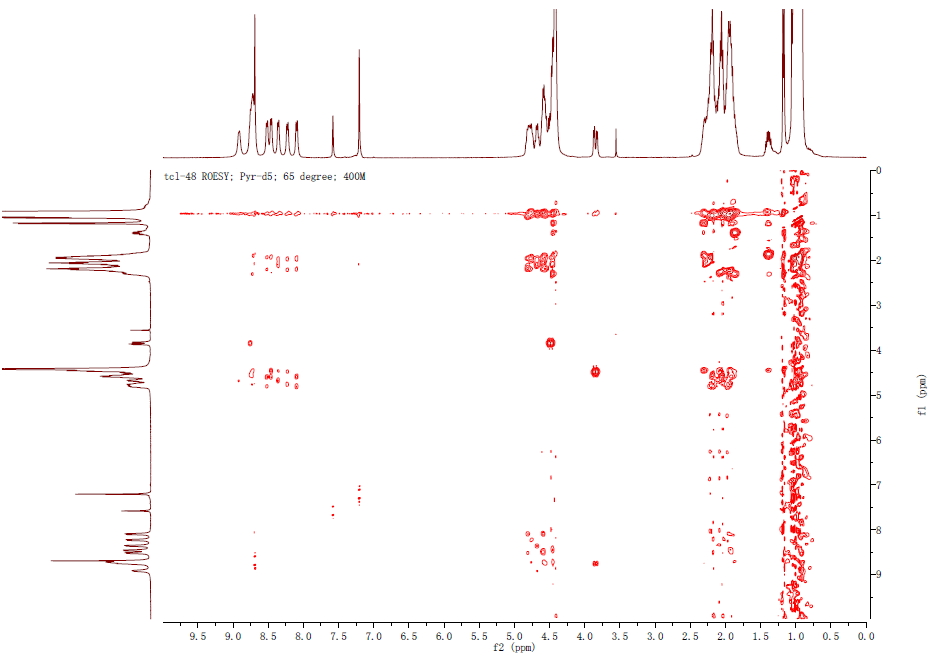


**Figure S7**. ESIMSMS spectrum of clausenlanin A (**1**)


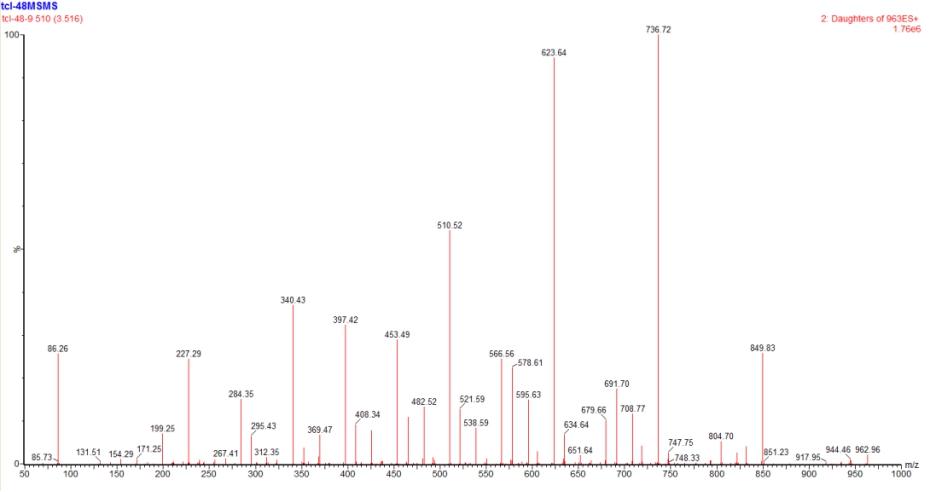


**Figure S8**. HRESIMS spectrum of clausenlanin A (**1**)


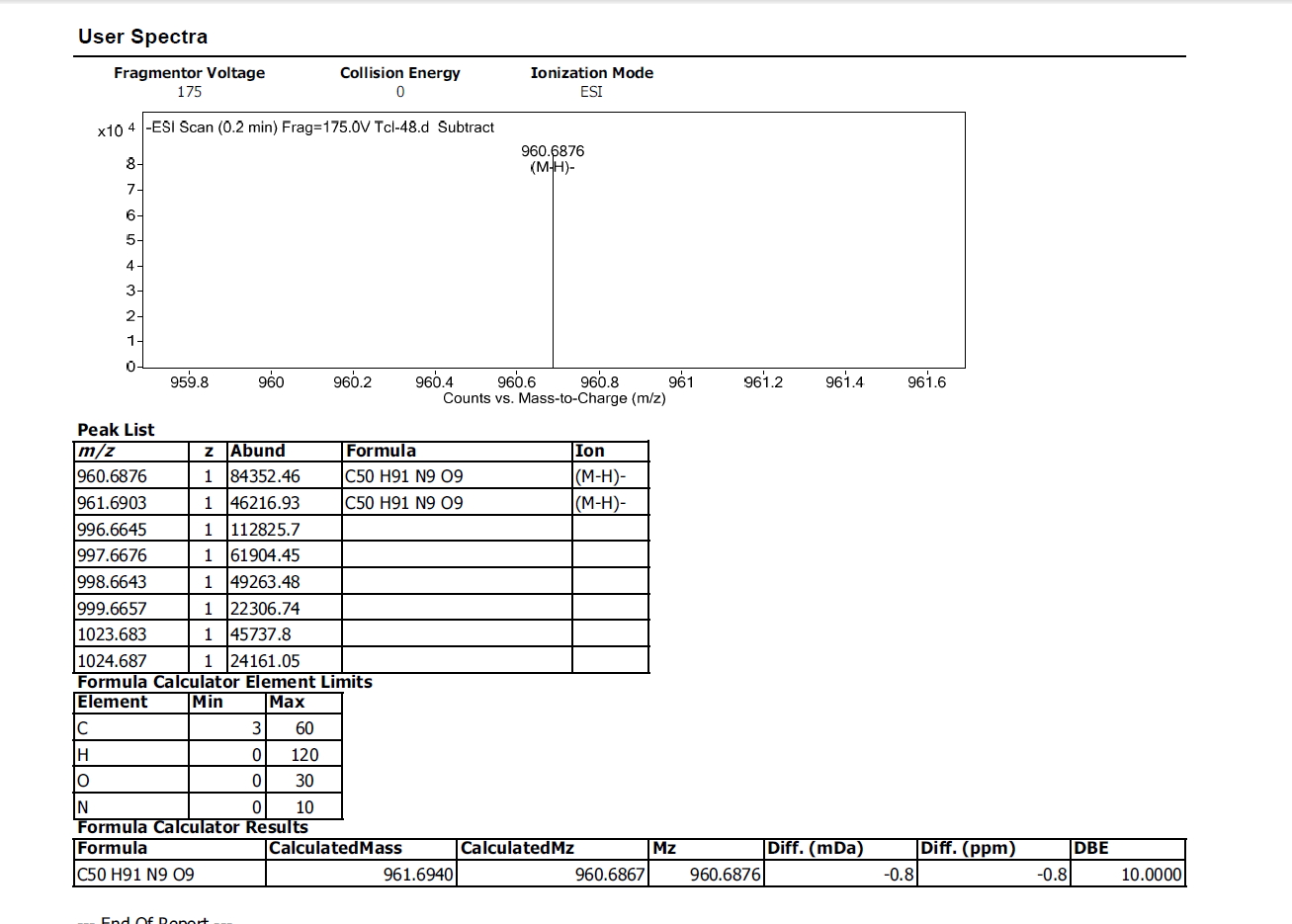


**Figure S9**. IR spectrum of clausenlanin A (**1**)

**Figure S10.** UV spectrum of clausenlanin A (**1**)


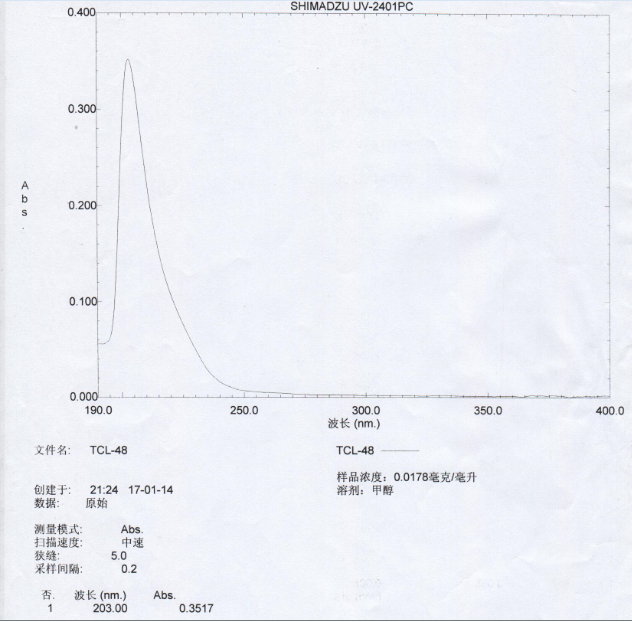


**Figure S11**. CD spectrum of clausenlanin A (**1**)


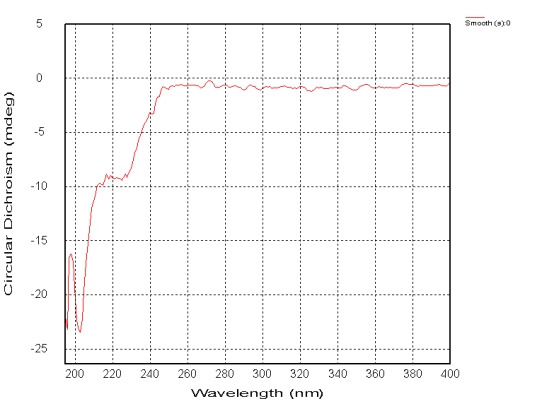


**Figure S12**. [α]^D^ spectrum of clausenlanin A (**1**)


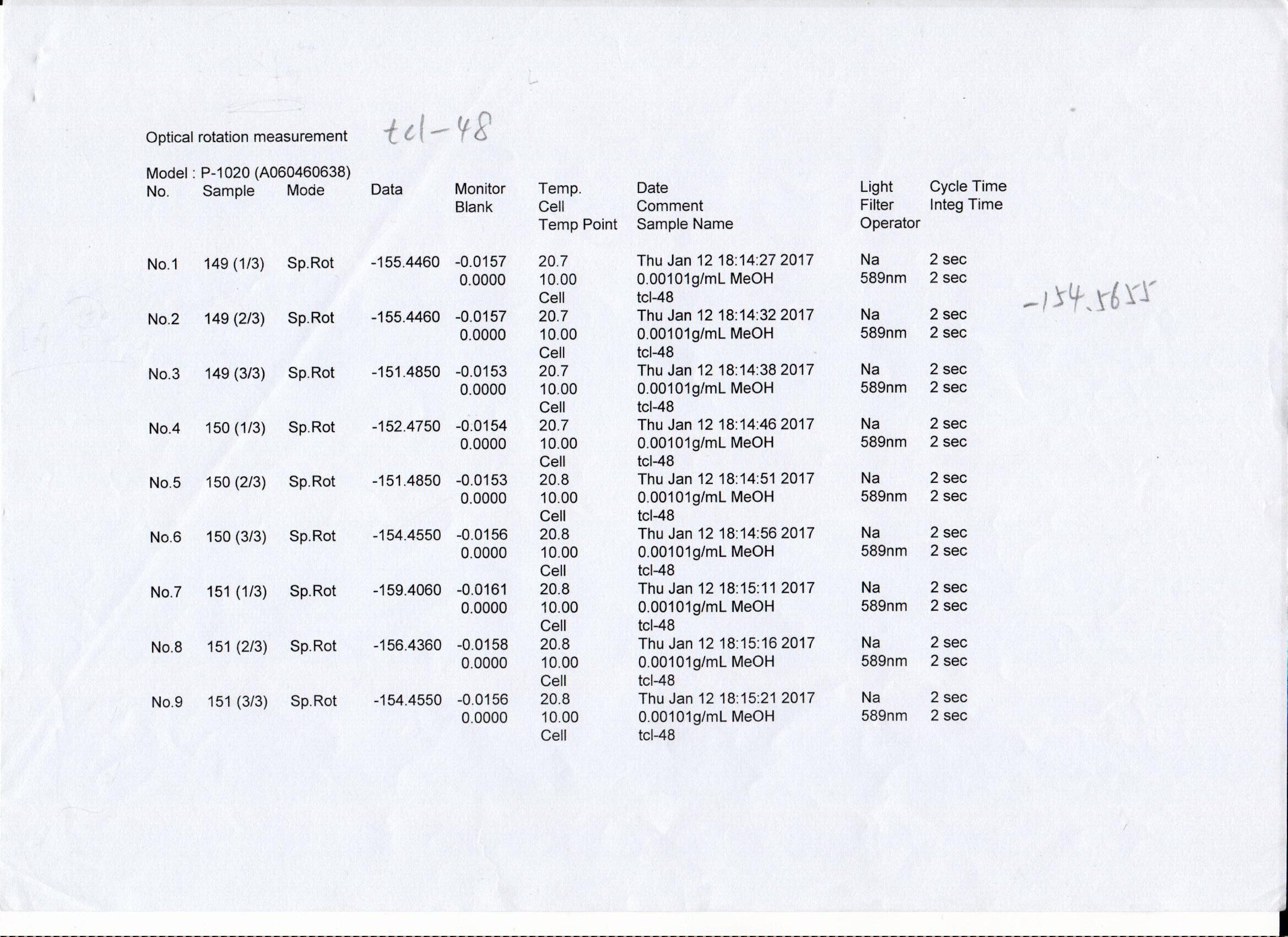


**Figure S13**. ^1^H NMR spectrum of clausenlanin B (**2**)


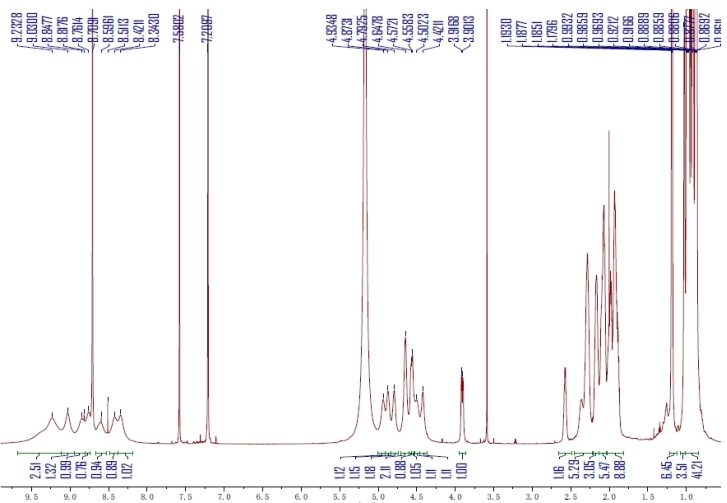


**Figure S14**. ^13^C NMR spectrum of clausenlanin B (**2**)


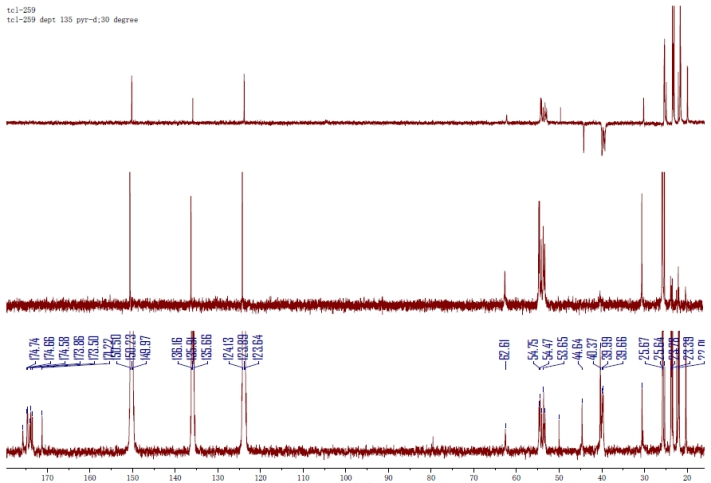


**Figure S15**. HSQC spectrum of clausenlanin B (**2**)


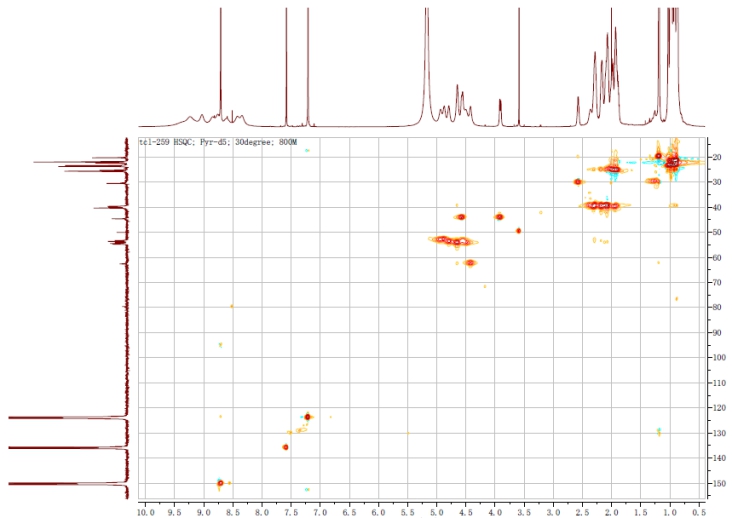


**Figure S16**. ^1^H-^1^H COSY spectrum of clausenlanin B (**2**) at 65 °C


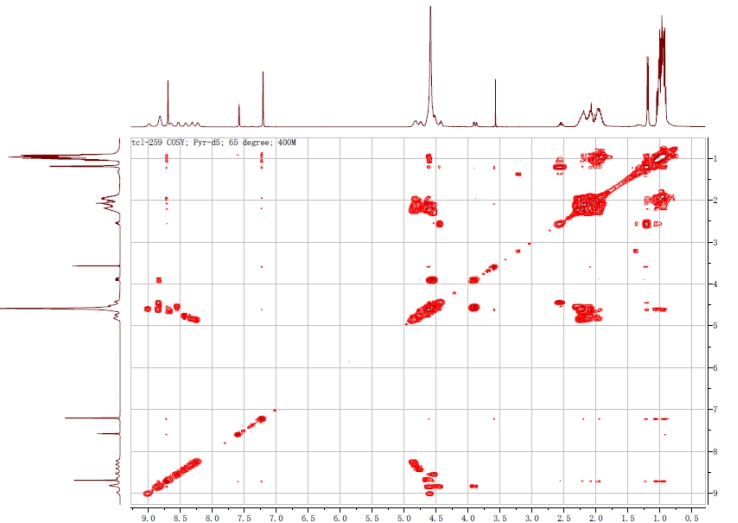


**Figure S17**. ^1^H-^1^H COSY spectrum of clausenlanin B (**2**) at 30 °C


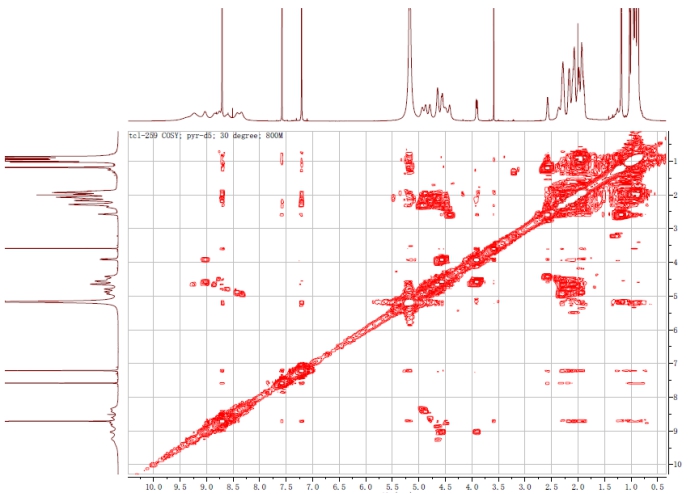


**Figure S18**. HMBC spectrum of clausenlanin B (**2**)


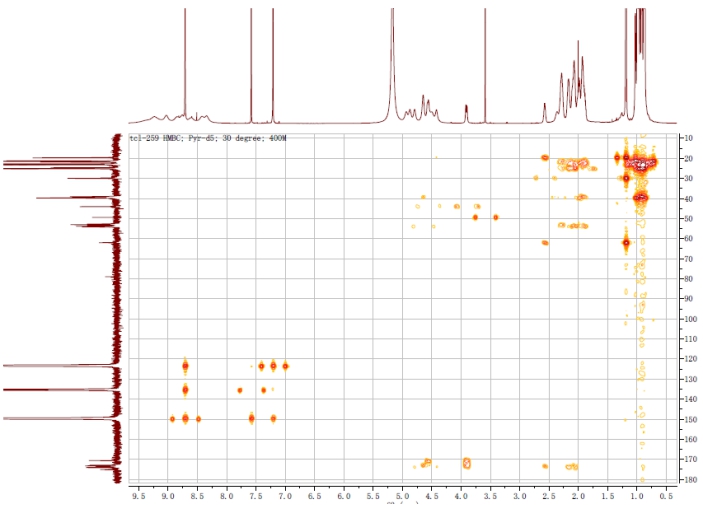


**Figure S19**. NOESY spectrum of clausenlanin B (**2**)


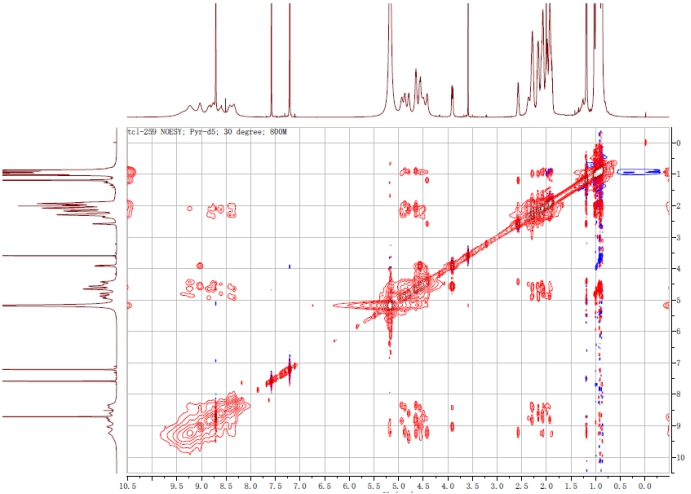


**Figure S20**. ESIMSMS spectrum of clausenlanin B (**2**)


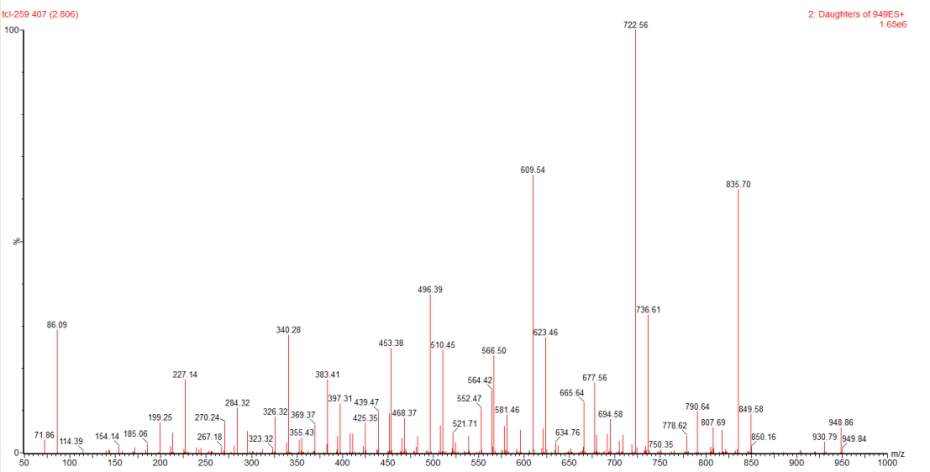


**Figure S21**. HRESIMS spectrum of clausenlanin B (**2**)


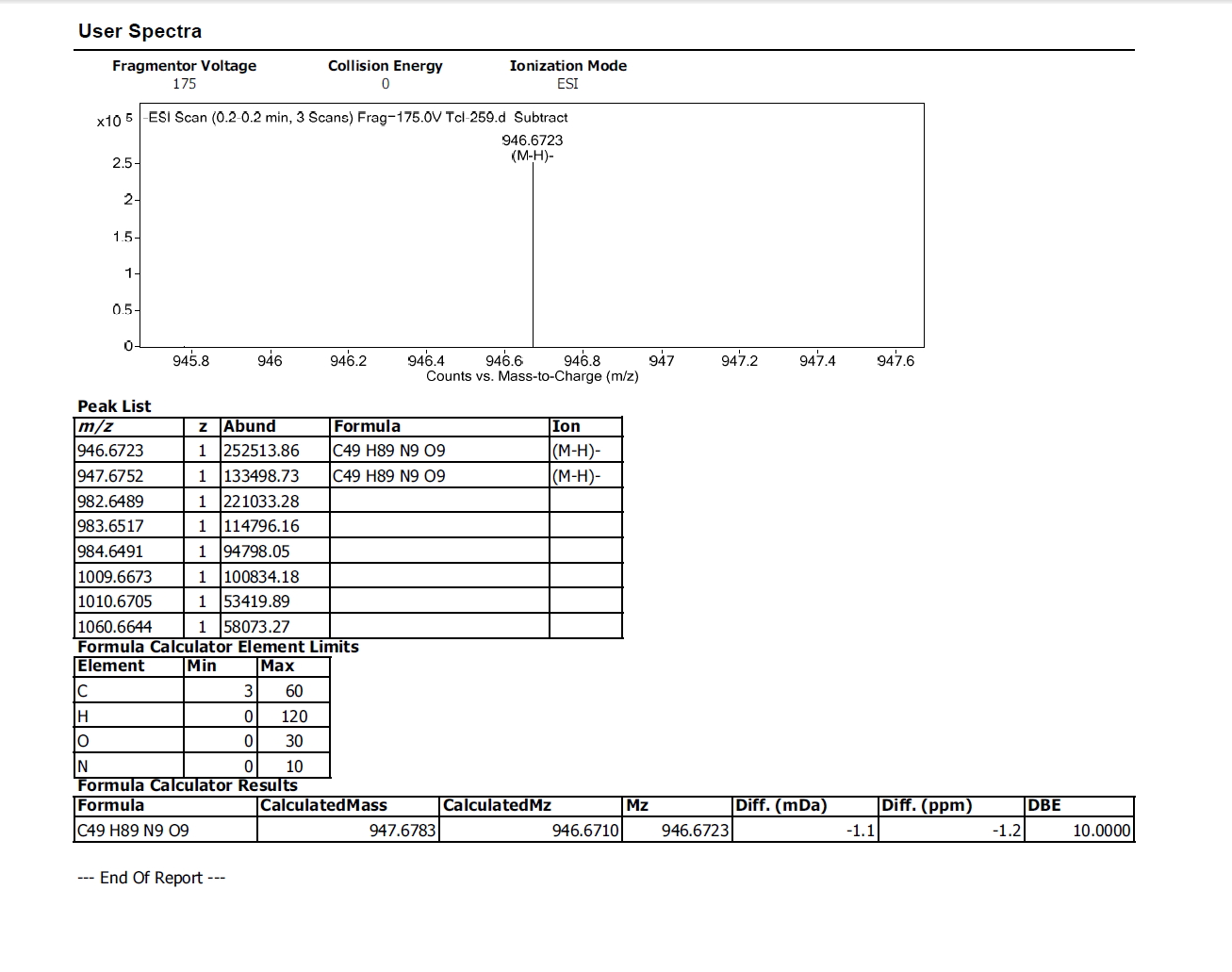


**Figure S22**. IR spectrum of clausenlanin B (**2**)

**Figure S23**. UV spectrum of clausenlanin B (**2**)


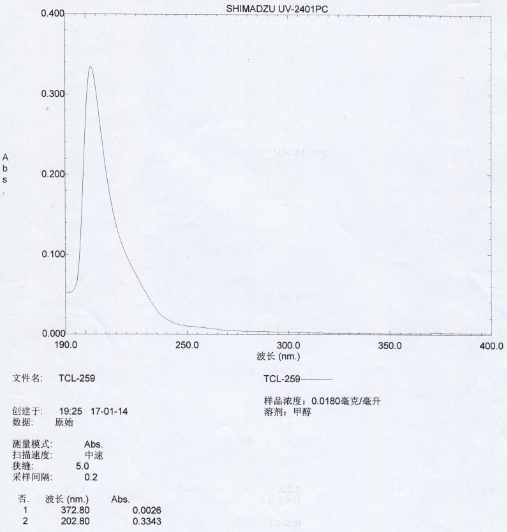


**Figure S24**. CD spectrum of clausenlanin B (**2**)


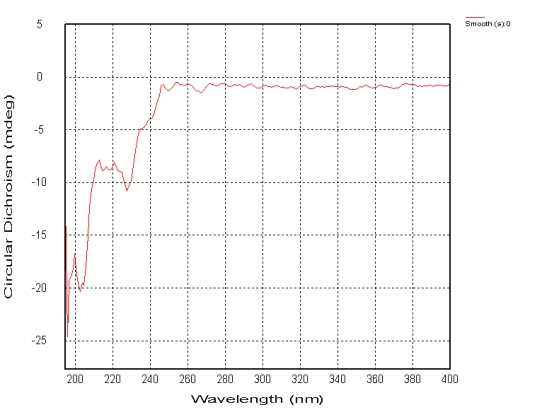


**Figure S25**. [α]^D^ spectrum of clausenlanin B (**2**)


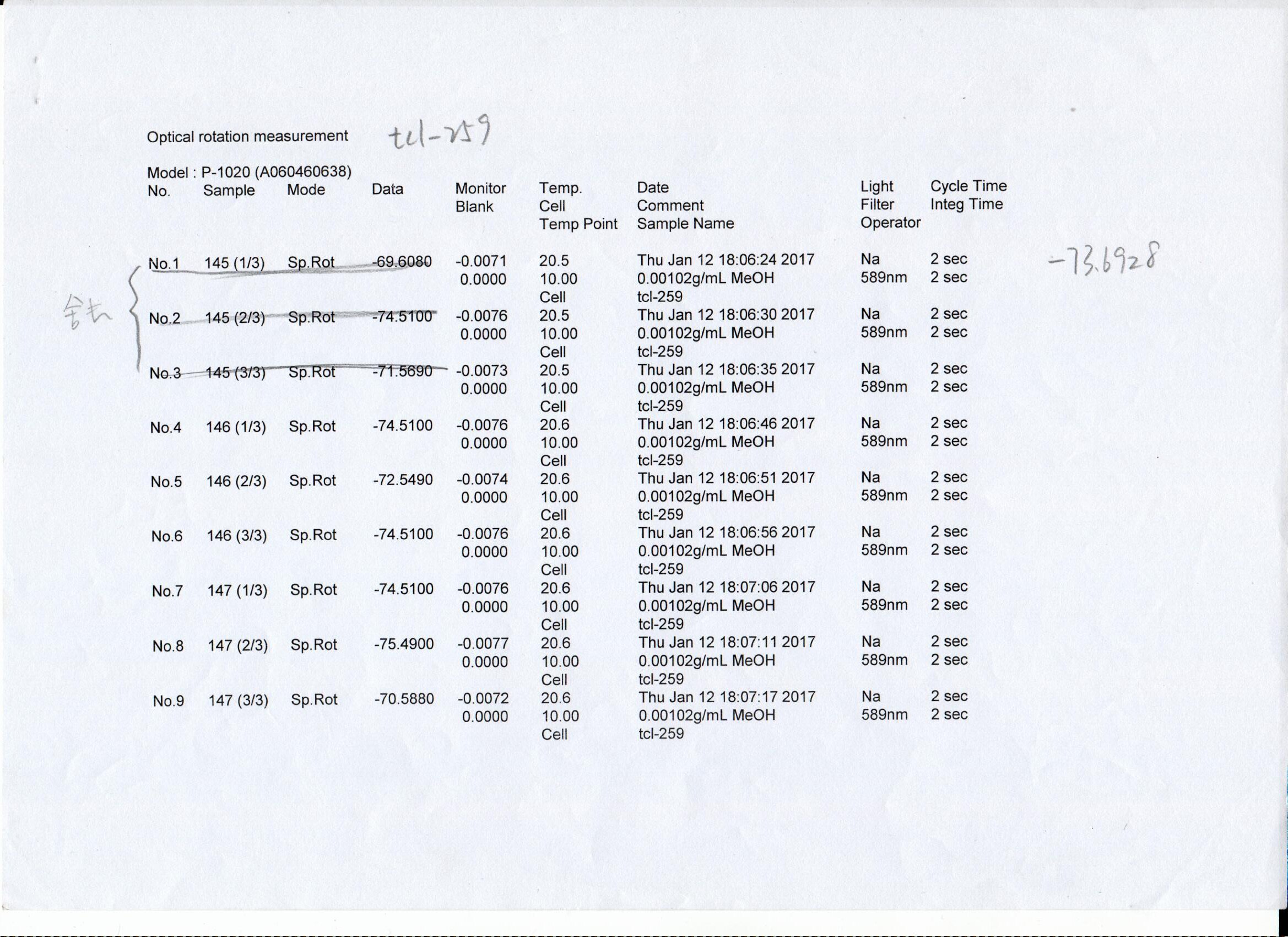


**Table S1**. Determination of the absolute configurations of amino acid residues in **1** and **2** by the advanced Marfey’s method

| Compound |  | *L*-Ile | *L*-Lue | *L*-Val |
| --- | --- | --- | --- | --- |
|  | [M-H]^-^ | 424 | 424 | 410 |
|  |  |  |  |  |
| **1** | t_R_ (min, *L*-FDLA derivatives) | 27.77 | 28.53 | / |
|  | t_R_ (min, *L*,*D*-FDLA derivatives) | 27.97; 39.93 | 28.83; 40.75 | / |
|  |  |  |  |  |
| **2** | t_R_ (min, *L*-FDLA derivatives) | / | 21.38 | 16.68 |
|  | t_R_ (min, *L*,*D*-FDLA derivatives) | / | 21.52; 35.16 | 16.74; 27.60 |
|  |  |  |  |  |
